# Evaluation of the acute flaccid paralysis (AFP) surveillance system in Mwenezi district, Masvingo, 2018: a descriptive study

**DOI:** 10.1186/s13104-018-3981-6

**Published:** 2018-12-10

**Authors:** Morgen Muzondo, Amadeus Shamu, Gerald Shambira, Notion Tafara Gombe, Tsitsi Patience Juru, Mufuta Tshimanga

**Affiliations:** 1 Department of Community Medicine, University of Zimbabwe, Harare, Zimbabwe; 2 Ministry of Health and Child Care, Masvingo Provincial Medical Directorate, Masvingo, Zimbabwe

**Keywords:** AFP, Mwenezi, Zimbabwe, Surveillance

## Abstract

**Objectives:**

Poliomyelitis is an infectious disease caused by the polio virus which affects mostly young children below the age of 15 years. For surveillance children with acute flaccid paralysis (AFP) are tracked. In Zimbabwe every district should report two cases per 100,000 population of children under the age of 15 years old. In 2017, Mwenezi district failed to detect any AFP cases. We therefore evaluated the AFP surveillance system in Mwenezi district. We conducted a surveillance system evaluation using the updated Centers for Disease Control guidelines for evaluating public health surveillance systems. We interviewed health workers in Mwenezi district and looked at AFP records from January to December 2017.

**Results:**

The main reasons for failure to report a case in 2017 were the vastness of the district with bad road networks as well as lack of a dedicated vehicle to carry out EPI outreach activities. About a quarter, 24%, of the health workers did not know the specimen that is used in AFP diagnosis. The AFP surveillance system in Mwenezi district was performing poorly due to lack of active search of cases in the community caused by disruption of EPI outreach activities.

## Introduction

Poliomyelitis is an infectious disease caused by the polio virus which affects mostly young children below the age of 15 years [1]. The polio paralysis affects usually one side of the body and is described as “acute flaccid paralysis” (AFP) [2].

Globally, polio cases have decreased by over 99% since 1988, from an estimated 350,000 cases in more than 125 endemic countries then, to 37 reported cases in 2016 [1].

In sub-Saharan Africa, the vast majority of global polio cases were found in Nigeria [3]. Nigeria however reported its last case of polio in 2014 [3]. Africa has remained polio free since 2014 [4].

Zimbabwe was declared polio free in 1999, but the threat of polio has remained due to the cases which were identified in Namibia and Botswana [5]. Certification of polio free status in Zimbabwe can only be done after demonstrable evidence that there are no polio cases in the Africa region [6].

**Fig. 1:**
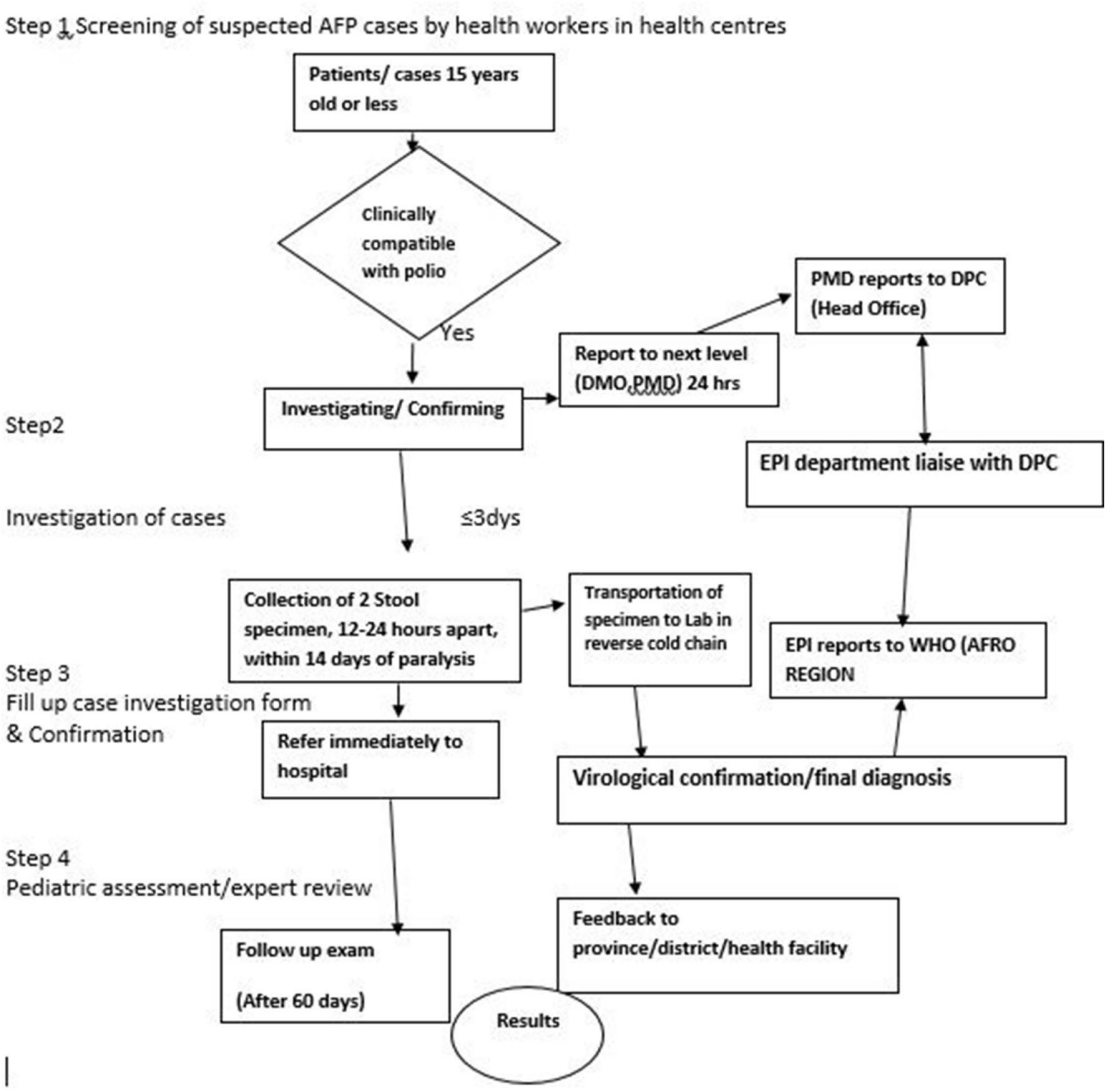
AFP surveillance flow diagram

The AFP surveillance encompasses detection of enterovirus from stool of children under the age of 15 years. The quality of acute flaccid paralysis (AFP) surveillance is based on the ability to detect at least one case per year of non-polio AFP for every 100,000 children under the age of 15 years [7]. In Zimbabwe the target has been increased to two cases per 100,000 children under 15 years of age as it still in the pre-eradication phase [8]. After a case is suspected, the District Medical Officer and the Provincial Medical Directorate (PMD) are notified within 24 h who also notifies the head office EPI Unit (Fig. 1).

In 2017, Mwenezi district in Masvingo province reported zero cases out of a target of four cases per year. Failure to detect AFP cases can result in wild type polio spreading. We evaluated the AFP surveillance system in Mwenezi district in order to identify reasons for its poor performance and make recommendations for improvement.

## Main text

### Study design

We conducted a surveillance system evaluation using the updated Centers for Disease Control guidelines for evaluating public health surveillance systems in March 2018.

### Study setting and population

We conducted the study in Mwenezi district of Masvingo Province. Mwenezi district has a population of 175,154 (i.e., according to 2012 census) and 50.2% are children below 15 years old. Mwenezi district has 24 health facilities and two are hospitals. All the health facilities offer immunization services. We interviewed health workers working in clinics and at hospitals in Mwenezi district and reviewed records of patients attended from 01/01/2017 to 31/12/2017.

### Sample size

Using the formula z^2^p(1 − p)/d^2^; based on a study done in Gokwe north in 2015 by Makoni et al. where a knowledge level among health workers was found to be 35%, at 95% confidence level, and power of 90%, and a non-response rate of 10%, a sample size of 87 participants were recruited into the study.

### Sampling

We purposively selected the two hospitals into the study as they are the largest institutions serving a greater population. Each hospital had 26 participants selected. The participants were randomly selected from the departments on those health workers who were found on duty on the day of the interview.

Systematic random sampling was conducted to come up with 12 clinics. At each clinic 3 health workers were randomly selected for the interview to make a total 36 participants.

Key informants, who are the District Medical Officer, Matron, District Nursing Officer, and laboratory scientists in the district, were purposively recruited into the study.

### Data collection methods

We used interviewer-administered questionnaires for health workers in Mwenezi district to assess knowledge on the AFP surveillance system and the system attributes (simplicity, acceptability, sensitivity, flexibility, timeliness, stability, representativeness and cost of running the system). Key Informant Interviews were conducted on the District Medical Officer, the District Nursing Officer, the hospital matrons and the community nurses to find out how the system works and to find possible reasons on not meeting the set targets. A checklist was also used to objectively assess the availability of the records and tools used in AFP surveillance.

### Data processing and analysis

Epi Info7™ was used for data analysis to generate means, proportions and frequencies and a 4 point Likert scale was used to assess the knowledge level.

## Results

We successfully recruited 87 participants into the study and seven key informants. All the health workers (n = 87) interviewed were nurses. The majority (i.e., 69%) were females. Most of the respondents (i.e., 60%) were registered general nurses. The median years in service were 9 years 9 (Q_1_ = 3; Q_3_ = 14) (Table 1).

**Table 1:** Demographic Characteristics of study participants in the AFP surveillance for Mwenezi district, 2018

Characteristic	Frequency n = 87	Percentage
Nurses	87	100
Gender		
Female	60	69
Males	27	31
Designation		
Registered general nurse	52	60
Primary care nurse	20	23
Nurse midwife	15	4
Median in service, years	9 (Q_1_ = 3;Q_3_ = 14)	

### Knowledge of the AFP surveillance system

Among the respondents, 95% (i.e., 83/87) knew the abbreviation AFP, and 55% (i.e., 39/87) knew the target age-group while only 6% (i.e., 5/87) knew the 60 day follow up after an AFP case is detected. Overall knowledge level was good among 52% of the participants (i.e., 45/87) (Table 2).

**Table 2:** Knowledge levels of participants in the AFP surveillance for Mwenezi district, 2018

Knowledge	Frequency n = 87	Percentage
General knowledge		
Poor	5	6
Fair	35	40
Good	45	52
Very good	2	2
Knew the abbreviation AFP in EPI surveillance	84	95
Aware of the specimen used for diagnosis	66	76
Knowledge of targeted age group monitored	39	55
Knowledge of the cold chain	77	89
Awareness of the 60 days follow up after an AFP case	5	6

### Simplicity of the AFP surveillance, Mwenezi district, 2018

Thirty-four percent (34%) of the respondents (i.e., 30/87) had ever encountered an AFP case and filled in the notification forms. Five out of the thirty reported taking 20 to 40 min to complete the forms and twenty five reported taking between 10 and 20 min while none reported taking more than 40 min. Five out of the thirty were observed filling in the form and it took them between 10 and 20 min the complete the form making the system simple.

### Stability of the health facilities on the AFP surveillance, Mwenezi district, 2018

Case definitions charts were available at all facilities visited and these were displayed in outpatients departments. The district relies on the district hospital EPI vehicle to transport stool specimens and forms. All the 14 health facilities had a working cellphone to relay data. Computers and internet connectivity were available at 19% (i.e., 3/14) of the facilities. Only 17% (i.e., 15/87) of the nurses were trained in AFP surveillance.

Few nurses, 17% (i.e., 15/87) were trained in AFP surveillance system. All the 14 facilities failed to carry out EPI outreach services. The district’s has one EPI vehicle, which was off the road due to lack of tires. The road network in the district to all facilities is bad.

### Usefulness of the AFP surveillance, Mwenezi district, 2018

The majority, 97% (i.e., 84/87 of the respondents reported that the AFP surveillance was useful. All the 14 facilities reported that the data should be used locally. However 43% (i.e., 6/14) reported using the AFP data for local public health actions like campaigns and community awareness. The majority of the facilities, 86% (i.e., 12/14) reported having meetings to discuss AFP data but however the minutes seen showed that the meetings were combined with other health surveillance conditions.

### Discussion

We found out that the AFP surveillance system in Mwenezi district was not sensitive and timeous as it failed to report a single case in 2017 despite the good knowledge they had. Failure of the surveillance system to pick cases can lead to wild type poliovirus circulating in the communities. The sensitivity was threatened by the vastness of the district with bad road networks as well as lack of a dedicated vehicle to carry out EPI outreach activities. Lack of a dedicated EPI vehicle can lead to failure by the district to do outreaches as well supportive supervision by the EPI program managers. Active search was not done and the surveillance was only focusing on those children who reported to the health facilities with other illnesses. This is however contrary to Saravoye et al. in Zvimba district, 2014 who found out that the system was sensitive [9].

We also found that about a quarter (i.e., 24%) of the health workers did not know the specimen used for diagnosis in AFP surveillance. Failure to collect two stool specimens within 24–48 h apart leads to stool inadequacy and failure to submit the specimen to the reference laboratory within 72 h leads to reduced chances of isolating the virus [10]. This is similar to findings by Bangure et al. in Sanyati where most of the health workers interviewed did not know the specimen used for diagnosis and they thought it was blood is used for diagnosis and this is detrimental as will lead to false negative results [10].

We also found that although the knowledge level of health workers in Mwenezi district on the AFP surveillance system was good, almost half of the health workers knew the target population to be screened. This is consistent with study findings in Gokwe north district, Zimbabwe, 2015 by Makoni et al. in an AFP surveillance study, where some health workers were unaware of the targeted age group [11]. This lack of knowledge on the target population means that some cases are missed leading to underreporting and spread of the disease in the communities.

On simplicity, although the system was failing, few people (i.e., 10%) reported that completion of the form was complicated while three quarters reported that the completion of the notification form was simple. The majority of the respondents reported that they needed training on filling in the notification form. Similar findings were reported by Saravoye et al. Zvimba district, Zimbabwe who concluded that the system was simple [9]. However, this was contrary to findings by Chimamise et al. in Mberengwa where completion of forms was found to be time consuming [12].

On stability, the AFP surveillance system in Mwenezi district was good since all the health facilities had a functional cellphone, which can be connected to internet through their network providers. However, some facilities were difficult to reach due to bad roads. This is similar to findings by Bangure et al. in 2013 in an AFP surveillance system evaluation in Sanyati district where the road network was poor and some clinics were inaccessible by road [10]. The system could however be threatened by the fact that they rely on the district hospital vehicle for transportation of specimens which in 2017 had no tyres for greater part of the year.

Most (97%) of the respondents perceived the AFP surveillance system as useful. When health workers perceive the system to be useful, they are more likely to participate constructively and diligently. All the health facilities had plotted graphs (zero reporting) and most of the facilities were holding weekly AFP surveillance meetings. However, the majority of the facilities were not using data at local level for public health action. No campaigns were done in the district and active search in the community was not being carried out. This can lead to missing cases in the communities. Similar findings were noted by Bangure et al. in Sanyati district, Zimbabwe, 2013 who concluded that if data is not used locally, the community would not benefit from the possible public health actions needed [10].

We conclude that the AFP surveillance system in Mwenezi district was performing poorly due to lack of active search of cases in the community caused by disruption of EPI outreach activities. However, there is a good foundation for improvement as the health workers have good knowledge of the system and they have the required resources to run the system.

We therefore recommended the training of health workers in AFP surveillance focusing on the target population as well as active case finding in the community. We also recommended planned advocacy meetings with community leaders on the importance of reporting to health facilities children who have symptoms. We also recommended additional use of motorized environmental health technicians in specimen collection to all the health facilities. This will help to reduce reliance on one EPI vehicle.

## Limitations

Our study had some limitations. There was recall bias in some participants in recalling when they were last trained in AFP surveillance. To counter this we limited the time to trainings done in the last 24 months. We conducted our study in a rural district and the results of our study may not be generalizable to urban districts.

## Abbreviations

AFP: acute flaccid paralysis
CDC: Centers for Disease Control
EPI: Expanded Programme of Immunisation
FETP: Field Epidemiology Training Programme
PMD: Provincial Medical Directorate
